# Experiences of Physical Activity, Healthy Eating and Quality of Life During and Following Pregnancy in Overweight and Obese Postpartum Women

**DOI:** 10.1007/s10995-023-03684-7

**Published:** 2023-06-14

**Authors:** Stephanie J. Hanley, Ian Varley, Craig Sale, Kirsty J. Elliott-Sale

**Affiliations:** 1 Institute of Applied Health Research, University of Birmingham, Birmingham, United Kingdom; 2 Sport Health and Performance Enhancement Research Centre, School of Science and Technology, Nottingham Trent University, Clifton Campus, Clifton Lane, NG11 8NS Nottingham, England; 3 Department of Sport and Exercise Sciences , Manchester Metropolitan University Institute of Sport, Manchester, United Kingdom

**Keywords:** Pregnancy, Postpartum, Physical activity, Diet, Lifestyle intervention

## Abstract

**Objectives:**

This retrospective study explored the experiences of women with overweight or obesity regarding physical activity, diet and quality of life leading up to, during, and following pregnancy.

**Methods:**

A qualitative descriptive design was adopted, whereby data collected through semi-structured interviews were analysed using thematic analysis. Throughout the interviews, individuals were asked to describe their barriers to a healthy lifestyle during and following pregnancy.

**Results:**

Ten women (34.5 ± 5.2 years old, BMI 30.4 ± 3.5 kg·m^− 2^) who were between 12 and 52 weeks postpartum participated. A range of themes were identified when discussing barriers to physical activity and healthy eating during and following pregnancy. For example, *tiredness*, especially in the third trimester of pregnancy, and a lack of *support* at home, was often cited as preventing engagement in exercise and healthy eating practices. A lack of *convenience* when attending exercise classes, *medical complications* following the birth and the *cost* of attending pregnancy-specific classes were identified as barriers to exercise engagement. *Cravings* and *nausea* were identified as barriers to healthy eating during pregnancy. Quality of life was positively associated with exercise and healthy eating, whilst a lack of sleep, loneliness and a loss of freedom since the baby had arrived negatively influenced quality of life.

**Discussion:**

Postpartum women with overweight and obesity experience many barriers when attempting to engage in a healthy lifestyle during and following pregnancy. These findings can be used to inform the design and delivery of future lifestyle interventions in this population.

**Supplementary Information:**

The online version contains supplementary material available at 10.1007/s10995-023-03684-7.

## Significance

*What is Already Known on this Subject?* Pregnant and postpartum women experience a multitude of barriers when attempting to engage in a healthy lifestyle.

*What this Study adds?* Until now, investigations into barriers to participation in a healthy lifestyle in overweight and obese pregnant and postpartum women have been lacking. Akin to normal weight women, women with a BMI > 25 kg/m^2^ experience many barriers to a healthy lifestyle during and following pregnancy. In this exclusive overweight and obese population, medical complications was the most cited barrier to postpartum exercise engagement. These results will be considered when designing future postpartum lifestyle interventions.

**Supplementary Information:**

The online version contains supplementary material available at 10.1007/s10995-023-03684-7.

## Introduction

Over half of the women of childbearing age in most developed countries are either overweight (body mass index (BMI) 25-29.9 kg∙m^2^) or obese (greater than 30 kg∙m^2^). (NHS Digital, 2017) Pregnancy can result in additional increases in BMI; for example, Johnson et al. (2013) showed that 73% (from a sample of 8293) of women gained weight in excess of the Institute of Medicine (IOM) guidelines. (Institute of Medicine (US) and National Research Council (US) Committee to Reexamine IOM Pregnancy Weight Guidelines, 2009; Johnson et al., 2013) In comparison to normal weight women, women who are overweight or obese are more likely to experience excessive gestational weight gain (GWG), (Deputy et al., 2015) which can result in adverse outcomes, including large for gestational age offspring, hypertensive disorders and a higher risk of caesarean section. (Johnson et al., 2013) The postpartum period is often defined as the 12 months after childbirth, during which time the weight gained during pregnancy should be lost. Women, especially those who experience excessive GWG (Nehring et al., 2011), often experience weight retention long beyond the postpartum period and enter subsequent pregnancies with higher BMI’s. (Kirkegaard et al., 2015)

Despite increasing evidence for the benefits of a healthy lifestyle during and following pregnancy on positive short- and long-term birth outcomes, (Aviram et al., 2011; Zhang & Ning, 2011) physical activity (PA) levels tend to decline, and diet quality, referred to as the balance between the consumption of healthy (e.g., wholegrains, fruits, vegetables) and unhealthy foods (e.g., sugar, sodium, saturated fats; (Guenther et al., 2013; World Health Organisation, 2018), worsens, especially in women with overweight and obesity (Moran et al., 2013). Often, PA levels remain reduced long into the postpartum period (Engberg et al., 2012), which may be associated with the decrease in perceived quality of life (QoL) after childbirth (Martínez-Galiano et al., 2019).

Previous research investigating the barriers to PA engagement during pregnancy have revealed a combination of intrapersonal and interpersonal barriers, including nausea and body shape changes, physical pain, a lack of knowledge about how to exercise safely whilst pregnancy and a lack of guidance from healthcare professionals (Coll et al., 2017). In the postnatal period, there are several shared and unique barriers to PA engagement, which include a lack of time, fatigue, and childcare responsibilities. (Cramp & Bray, 2011)

Pregnancy symptoms such as nausea and food aversions, low socioeconomic status, and psychological factors such as levels of eating restraint in anticipation of GWG have been shown to limit diet quality in pregnant women. (Anderson, 2001; Doyle at al., 2016) Little work has explored specific barriers to healthy eating in postpartum women, but in a low-income setting, breastfeeding mothers cite being too busy to shop for fresh ingredients and prepare nutritious meals, and more time is spent caring for the baby despite the knowledge that a healthy diet positively affects overall health. (MacMillan Uribe & Olson, 2018)

Although the knowledge base surrounding potential barriers to following a healthy lifestyle during and following pregnancy has expanded in recent years, there remains a dearth of information related to barriers to participation in overweight and obese participants and further work is required to understand these women’s experiences during and following pregnancy. It may be that overweight and obese women experience unique challenges, which are weight-related, that limit their ability to adopt mainstream lifestyle interventions. Indeed, a number of postpartum lifestyle interventions in overweight and obese populations have proven ineffective in promoting behaviour change (Heppner et al., 2011; Skouteris et al., 2012; Vesco et al., 2012) and significantly reducing BMI. (Østbye et al., 2009; Walker et al., 2012) A comprehensive understanding of the barriers preventing overweight and obese women from maintaining a healthy lifestyle during and following pregnancy is crucial in order to guide the design and delivery of future lifestyle interventions, with the aim of promoting long-term health, appropriate GWG and postpartum weight loss. Thus, the aim of this study was to examine the experiences of overweight and obese women regarding PA, diet and QoL leading up to, during, and following pregnancy. The results can be used to inform the design and delivery of lifestyle interventions in the same population.

## Methods

This study was part of a PhD programme of work which sought to gain an understanding of participants’ experiences before, during and following pregnancy, through rich descriptions. Furthermore, the study aimed to generate results that would be available to practitioners to underline practical applications and to inform the design of future intervention-based research studies. As such, the Qualitative Descriptive approach described by Sandelowski (2000) was adopted, underpinned by an interpretivist perspective. The research team explored individuals’ unique experiences leading up to, during and following pregnancy whilst recognising that experiences are socially constructed and based on individual interpretation.

### Participants

Potential participants were purposefully sampled based on criterion-i sampling to ensure in-depth accounts and thus sufficient information to address the research questions. (Patton, 2002) Participants were invited to take part if they were primiparous, had a singleton pregnancy and were between 12 weeks and 52 weeks postpartum. Given that women attend a six to eight week check with a general practitioner to determine if normal PA can be resumed following childbirth, twelve weeks postpartum was deemed sufficient time to allow individuals the opportunity to experience and identify postpartum barriers to a healthy lifestyle and to allow sufficient recovery time from childbirth, especially in those who had had a caesarean section. At the time of study participation, participants also had to have a BMI of greater than 25 kg·m^− 2^.

### Procedure

The study was conducted in accordance with prevailing ethical principles and reviewed by an Institutional Review Board. Following ethical approval, study advertisements and posters were placed on notice boards in the community and on various social media sites (e.g., Facebook, Twitter, Mums Net). Two adverts were placed on social media over a 14-day period and the study was also advertised through internal university staff email distribution lists and word-of-mouth, whereby colleagues shared the information with friends and family. Potential participants contacted the research team directly to indicate their interest and were provided with more detailed information regarding the study. Through snowball sampling, several participants identified other potential individuals who fulfilled the inclusion criteria and passed on the study details to them such that communication with the research team was instigated by the potential participant to ensure that they did not feel that they had to participate or felt compelled to reply in a certain manner. There were no study dropouts or refusals and, unfortunately, detail was not collected on numbers screened but who were then not recruited.

### Data Collection

Participants were provided with detailed verbal and written explanations of the study and informed consent was obtained. Interviews were broadly structured as a life-history interview, such that participants were encouraged to share stories from throughout their life and, where possible, placed these stories within specific historical life stages (e.g., childhood, stage of pregnancy). (Smith & Sparkes, 2017) This approach allowed participants to take control of the interviews and to position their experiences along the time course of their pregnancies and into the postpartum period.

Prior to conducting any interviews with the intended participants, an interview guide was piloted, which allowed the interviewer to become familiar with the interview questions. Following the pilot interview, the interview guide was revised such that introductory questions were included to address each of three main topics: PA, nutrition and opinions on the design of lifestyle interventions. For example, on the topic of PA the first question was, “When I say the words “physical activity” what comes to mind?” (Appendix 1). Prior to the formal interview, time was spent building rapport with the participants and the layout of the interview guide was slightly altered to gain a clearer understanding of overall, childhood, pregnancy and postpartum experiences when discussing each topic.

Interviews were conducted in a private room on a university campus or at the participant’s home by female researcher, SH who is experienced in qualitative interviewing and was conducting this work as part of her PhD. Participants were given the choice of where they wanted the interview to take place, which may have enabled them to feel more comfortable to speak openly and empowered in their interaction with the interviewer. (Elwood & Martin, 2000) All participants attended with their babies, and prior to the interview were informed about the wider programme of work and that findings would feed into the design and delivery of a postpartum lifestyle intervention. Data collection for the study was completed over a 4-month period, no repeat interviews were completed, and transcripts were not returned to participants for feedback. The interview guide contained questions about the delivery, views and experiences of PA and diet during childhood, stages of pregnancy and postpartum. Interviews ranged in length from 28 to 45 min (36 min 17 s ± 5 min 18 s), excluding the time spent building rapport with participants, prior to commencing audio recorded interviews which were transcribed verbatim.

### Data Analysis

Thematic analysis, based on the approach adopted by Braun and Clarke was completed. (Braun & Clarke, 2006; Braun et al., 2017) Familiarisation of the data, or transcripts, occurred through the process of immersion, which involved repeatedly reading the data and identifying any emerging specific patterns and meanings in the data. Following this, a detailed reading of each transcribed interview was carried out, highlighting potentially meaningful or interesting ideas and arranging them under different headings (termed codes; for example, ‘reduced physical activity during pregnancy’, ‘lack of dietary restraint’, ‘long-term weight issues’). Next, themes were developed which were interpretative and focused on aspects of the participants’ experiences, for instance of diet or PA or QoL. Coded data were arranged under themes derived from the data and relationships between the codes, themes and different theme levels (e.g., main overarching themes and sub-themes) were also developed. During and following the process of thematic analysis, themes were further refined to reflect all appropriate codes. Such refinement occurred initially at an individual researcher level and then independently by another member of the research team, and where necessary, any conflicts were discussed and resolved.

Investigator triangulation existed whereby, to confirm the findings and different perspectives, and to add breadth to the phenomenon of interest (Denzin, 1978), SH and KES coded the data independently and, following discussion, agreed on the final list of themes. Data source triangulation allowed for the selection of in-depth semi-structured interviews to elicit rich detail regarding personal experiences of PA, diet and QoL before, during and after pregnancy. To reduce confirmation bias, throughout the research process, SH and KES continually re-evaluated participants’ impressions and responses through ongoing discussion, and where necessary, involved other members of the research team. The interview guide was structured such that general introductory questions were asked first, followed by more specific questions regarding individuals’ experiences and barriers, to reduce question-order bias.

### Methodological Rigour

Smith and Sparkes (2017) have suggested that the quality of qualitative research should be judged using a relativist, rather than a criterion, approach. (Smith & Sparkes, 2017) Consequently, the nine proposals by Smith and Caddick (2012) that were applicable to the current study were employed; namely *substantive contribution, impact, comprehensiveness of evidence, coherence, catalytic and tactical authenticity, resonate and credibility and transparency*. (Smith & Caddick, 2012) Specifically, our aim was to produce a paper with *substantive contribution* towards the understanding of experiences of PA, diet and QoL in postpartum women with overweight and obesity; the justification for which is outlined throughout the introduction section. We believe that, through the engagement with a group of postpartum women where little research has been completed to understand their experiences, this work will have *impact* by stimulating ideas for future lifestyle intervention development and provide a basis for which practitioners can support these women to overcome barriers to a healthy lifestyle. It is hoped that *comprehensiveness of evidence* has been achieved by the extensive explanation of study findings and use of quotes. Furthermore, we have attempted to establish *coherence* by providing continuity between the research aims, data collection and analysis methods, and description of results.

This work has already fed into the development and delivery of a successful postpartum lifestyle intervention for women with overweight and obesity (paper in preparation), which likely indicates *catalytic and tactical authenticity.* By completing a pilot interview and engaging with women at different stages, we sought to achieve *credibility and transparency*. Finally, by providing a detailed overview of women’s experiences during and after pregnancy, and a list of suggested practical applications in the context of previous literature, we believe the results will *resonate* with our readers.

## Results

Ten participants (mean ± SD; 34.5 ± 5.2 years, BMI 30.4 ± 3.5 kg·m^− 2^, 7.1 ± 2.9 months postpartum) were recruited between January and March 2018. Participant characteristics and information regarding the provision of PA and dietary advice during and following pregnancy is displayed in Tables 1 and 2, respectively. After eight interviews there were no new themes being generated. To confirm that data collection had reached saturation, a further two interviews were conducted (Francis et al., 2009). Throughout the interviews, participants were asked to recount their experiences; in the years prior to, during and following pregnancy. Themes were organised and presented in line with the overall study aim to understand the barriers to PA, healthy nutrition and QoL during pregnancy and in the postpartum period in overweight and obese postpartum women. Tables 3 and 4 display the results regarding perceived barriers to PA and nutrition during pregnancy whilst perceived barriers in the postpartum period are presented in Tables 5 and 6. Table 7 displays findings regarding QoL.

**Table 1:** Participant demographics

Participant no.	Ethnicity	Level of education	Maternity leave status (Yes/No)	Breastfeeding status (duration)	Support (Yes/No) (Source(s))
1	White	Degree	Yes	Yes	Yes (Mother-in-law, partner)
2	White	Degree	No	No, stopped (10 months)	Yes (Husband)
3	White	Degree	Yes	No, stopped (7 months)	Yes (Husband)
4	White	Degree	Yes	Yes	Yes (Husband)
5	White	Advanced Diploma	Yes	Yes	Yes (Partner)
6	White	Degree	Yes	Yes	Yes (Husband)
7	Black Caribbean	Degree	Yes	Yes	Yes (Husband)
8	White	PhD	Yes	No, stopped (3 months)	Yes (Husband)
9	White	Level 3 Diploma	Yes	No, stopped (1 week)	Yes (Husband, parents)
10	White	Postgraduate Diploma	Yes	No, stopped (4 weeks)	Yes (Husband, parents)

**Table 2:** Physical activity and dietary advice providers

Participant no.	PA advice pregnancy?	Who?	PA advice postpartum?	Who?	Diet advice pregnancy?	Who?	Diet advice postpartum?	Who?
1	Yes	Midwife, GP	No		Yes	Midwife	No	
2	No		Yes	Personal trainer	No		No	
3	Yes	Health visitor	Yes	Health visitor	Yes	National Childbirth Trust	Yes	Internet
4	Yes	Personal trainer	Yes	Personal trainer	Yes	Personal trainer	Yes	Personal trainer
5	Yes	Community midwife	No		Yes	Community midwife	No	
6	No		No		No		No	
7	No		No		Yes	Health visitor leaflets	Yes	Health visitor leaflets
8	No		Yes	Midwife	No		No	
9	No		Yes	Midwife	Yes	Midwife/doctor	No	
10	No		Yes	NHS	Yes	NHS	No	

**Table 3:** Perceived barriers to PA during pregnancy

Barriers to PA (Pregnancy)		
Theme	Sub-theme	Example Code
Tiredness	Too tired	In the first trimester it’s so tiring, like you’re so exhausted for no apparent reason you just feel exhausted, so you have literally zero energy. (P05)
Support	Little advice	The only advice I’ve got is stuff that, well I know myself, or look on the internet and that sort of thing. (P03)
	Discouraged engagement	Stopped running in my second trimester because somebody made a comment to my husband… Should she be running? And I don’t know it frightened me. (P02)
Work	Work prevents class attendance	Swimming times for adults tend to be during the day… and when you work full time you can’t really get there. (P04)
Physical	Bigger and more cumbersome	Went swimming once, we basically just floated around because we were just two big whales together. We were huge. (P05)
	Nausea	Quite nauseous and probably only managed to go to the gym maybe once a week until probably week sixteen. (P01)
	Need toilet more often	And then basically I needed the toilet every time I, like running out of the class ever ten minutes, like oh god… So I just did pilates and a bit of yoga at home, that sort of stuff. (P01)
Convenience	Time of day [don’t like evenings]	You know I was in work by 7.30am leaving governor’s meetings at 6.30 at night. The last thing you want to do is go to the gym…Whereas before, I probably could have done it. But all I wanted to do was go home. (P04)
	Unable to locate classes	There was just nothing really available… couldn’t find anything that got me going either. (P08)
Health and safety	Fear of miscarriage	I think I was probably about five weeks pregnant or something when I found out so not that far gone, and then I waited, I basically stopped going to the gym at that point myself because I was really conscious about not exercising too much because I really didn’t want to lose the baby. (P05)
	Hockey contraindicated during pregnancy	So the physical activity I’d done before I was pregnant, I couldn’t carry on with. I mean you can play hockey when you’re pregnant, but it’s not a good idea to. (P04)
Time	Lack of time	So, what do you think stopped you from picking up anything new when you weren’t able to play hockey and cricket anymore? Possibly time a bit. (P09)
Cost	Too expensive	Paying for a gym membership is expensive and a lot of them tie you in. (P09)

**Table 4:** Perceived barriers to healthy nutrition during pregnancy

Nutrition Barriers (Pregnancy)		
Theme	Sub-theme	Example Code
Cravings	Crave (rubbish)	So, during the first trimester you do just crave absolute rubbish which I found quite surprising because you’d think that your body would want to have stuff that’s really nutritious and good for you. (P01)
	“Needed” salt	Just those first couple of weeks I just needed salt and crisps and paninis basically and hash browns. (P01)
Nausea	Morning sickness [better when eating]	I had really bad morning sickness, but it was sickness all the time and the only thing that would stop it was eating. So I just ate. (P02)
	Repulsed by meat & other foods	The sight of meat repulsed me and I was, I was in [supermarket name], picked up some like turkey mince and just started retching and had to run out of the shop. (P01)
Restraint	Not drinking, eating more	Because I wasn’t going out on the weekends and drinking wine, I was thinking actually, that’s loads of calories saved, it probably doesn’t matter if I have a bit of a treat. (P01)
	Having treats, no restraint	Before I had a little bit more self-restraint, but when I was pregnant I was like oh it doesn’t matter… I’m probably going to get a bit fat anyway. (P01)
Tiredness	Feel rubbish, eat crap	When I got tired would be grabbing something on the way home. (P04)

**Table 5:** Perceived barriers to PA in the postpartum period

Barriers to PA (Postpartum)		
Theme	Sub-theme	Example Code
Medical Complications	Episiotomy	She had to be delivered by forceps because her heart rate was dropping so they decided they needed to get her out pretty quick and obviously as they went to cut me to get the forceps in, because they’re pretty big, don’t ever look at them. They cut me to my back passage unfortunately, so I had to go straight into surgery to be stitched back together afterwards. (P04)
	Heavy bleeding	Experienced quite heavy bleeding during that time as well, so that’s particularly uncomfortable. (P01)
	Pelvic pressure	Very, very conscious… make sure I go for a wee beforehand. (P02)
	Reduced strength	Because usually, I use my stomach muscles you know, to like get up and I just couldn’t do it, so he had to come over and take the bar off and I had to sort of roll off the bench. (P01)
	Unfused stomach	Checking to see whether your muscles are fused, the doctor doesn’t check that. They just ask you questions. (P01)
	Back pain/pressure	Found it [at home exercise program] was putting too much pressure on my back. (P01)
	Body not ready/too heavy for return to exercise and sport	Because there’s no way after a year my body is ready to go back to playing hockey. (P04)
	Recovery from c-section	It took ages for my C-section scar to heal and yes a lot longer than other friends of mine seem to… I think they said I could exercise after twelve weeks but I took ten weeks before I could walk properly again, so I didn’t do any exercise for ages. (P10)
Convenience	Hard to get to classes	The one [exercise class] at the hospital it’s a faff getting to anyway when you’ve got an appointment in there. (P01)
	Issues with transport	Don’t have a car today and so it’s difficult. (P01)
	Inconvenient	If someone said to me oh there’s a baby class in [place name] or in the next village, I would probably go to it, but it’s the fact that they tend to be that little bit further away. (P03)
	Lack of parking	Do yoga and things like that but the parking is terrible so that would tend to put me off. (P03)
	Unable to locate appropriate classes	I know they do Pilates and yoga, but that to me is not enough. I want to do a proper workout. (P08)
Routine	Exercise second to baby’s needs	And I imagine that’s what most mothers would say, their eating and exercise is secondary to the baby basically. (P01)
	Baby’s lack of routine makes exercising difficult	When she was little, we weren’t quite sure of her routines and you wouldn’t be quite sure when you could take her out. (P03)
	Need a routine to incorporate exercise into	I think the main thing is that I need to get into a routine of doing regular exercise. (P06)
	More to do now- less time to exercise	If I really wanted to I could go out for a run while my husband baths my baby but I’m tired and I’ve got loads more jobs to do. (P02)
Support	Depression- loneliness	Not depressed and a bit crap. So when you are feeling like that, the last thing you want to do is go to the gym. Even if you know it will make you feel better. (P01)
	Nobody to exercise with	If I’m not going with someone am I going to be lonely? (P03)
	Lack of advice	The only advice I’ve got is stuff that, well I know myself, or look on the internet and that sort of thing. (P03)
Time	Lack of time	Just got a gym membership but it’s a lot harder to find the time to go. (P09)
Childcare	Lack of childcare	We’ve got no family nearby so getting someone to look after him while I go to the gym or something just can’t happen. (P06)
	No freedom	It’s just not having the freedom to just go and do a gym class whenever you want. (P01)
Tiredness	Too tired	I get to like 7pm I’m just like so exhausted from entertaining him all day. (P06)
Motivation and enjoyment	Not feeling up to it	Because there’s no way after a year my body is ready to go back to playing hockey. Well it would be ready to go back to playing hockey, but I would be frustrated that it wasn’t at the same level as it was before because I’ve had a year off. (P04)
	No motivation	I could do it every night if I had any motivation, but I have very little. (P06)
	Not enjoying it as much as before	(weakened pelvic floor) stops me enjoying it (exercise) as much as I used to enjoy it. (P02)
Cost	Too expensive	I’m on statutory maternity pay, so that’s another like barrier for me because it’s just like well I can afford to go to the gym because it’s like 10 pounds a month but I don’t know how much I’ll be able to go to the baby exercise classes. (P05)
Breastfeeding	Issue with breastfeeding	I don’t express, so there’s literally no one else to feed her other than me. (P05)
Confidence	Lack of confidence	I can go to a class for her because it’s easy because it’s her focus. But a class for me is a bit more oh not quite sure. (P03)

**Table 6:** Perceived barriers to healthy nutrition in the postpartum period

Nutrition Barriers (Postpartum)		
Theme	Sub-theme	Example Code
Time	No time to cook	Even though he’s eating food that we could eat, I just think once I’ve fed him my food is cold, he’s wanting then entertaining, so the time thing is a real like an issue in that sense. (P06)
	Less time to cook	He fills so much of my head at the moment and thinking about him and doing all the extra washing and extra responsibilities and jobs that come with having him, I struggle to fit time in thinking about food prepping and meals and stuff. (P09)
Tiredness	Eat crap, feel tired, feel more crap	Lack of routine and lack of motivation sometimes and just being tired and craving crap. (P09)
	Lack of sleep	But that (tiredness) just leads into like unhealthy eating habits because when I’m like up all night I just think basically how am I going to treat myself for doing this stint all night. (P06)
Routine	Eating second to baby’s needs	When I was looking after [baby’s name] all the time you’d live on toast or a sandwich or whatever and I needed some structure. (P02)
	Baby’s lack of routine	I’m hoping that as he gets bigger and as he gets into more of a routine then that will change. (P07)
	No routine	Lack of routine and lack of motivation sometimes. (P09)
Support	Need support [at home]	My husband and I we really need to support each other in it because if one of us does it doesn’t really work because you’re sort of living together and eating together. (P03)
	Someone else doing the shopping [no control over choices]	Because he does the bloody shopping he doesn’t always get everything that I want, he’ll get what he wants. So there is not necessarily enough stuff for me to eat and for me to think that’s what I would really like to eat and I can make something really healthy with that. (P05)
Motivation and enjoyment	Lack of motivation	So I can be quite lazy and so can my husband and If I say I can’t be bothered to cook we’ll just go for a takeaway or something. (P04)
Breastfeeding	Breastfeeding as an excuse to eat more	I was of the opinion that I was breastfeeding so it didn’t matter, calories didn’t matter because you were feeding for her… I would go for chocolate, crisps, doughnuts, all that kind of stuff and in my head I thought that was okay because I’m breastfeeding, using up the calories, but clearly not. (P04)
Restraint	Have treats when tired, no restraint	It is just having a few treats, especially when you’re tired. You kind of, you want a little bit of a chocolate hit for the energy. (P01)

**Table 7:** Influences on quality of life in the postpartum period

Quality of Life (Postpartum)		
Theme	Sub-theme	Example Code
Lifestyle	Exercise	I know when I have a decent amount of exercise it makes me feel better. (P03)
	Healthy eating	It affects my mood in a negative way if I don’t feel happy with what I’m eating. (P05)
Sleep	Lack of sleep	I am tired and I’m hungry, but I just felt really, really low, and like I looked at the symptoms and stuff and I am definitely a bit postnatal. And he’s (partner) like “babe you’re not you’ll be fine you literally just need some good sleep.” And then I had a couple of hours sleep and I woke up and I felt loads better. (P05)
Loneliness	Loneliness affecting mood	It’s lonely, and you get cabin fever and you’re staring at the same four walls. It’s hard. That was when, breastfeeding with her, it was hard, because I couldn’t go out. (P08)
Freedom	Lack of freedom	Because you can’t just nip out and go shopping and stuff, like before when I was off, before I had her, I would like go out with my friends and stuff and meet them for lunch and whatever and then I’d go off to town shopping or nip up to [place] to see my parents or that sort of thing, just go ahead and do whatever I wanted whenever I wanted. And now I can’t do that so that just makes it, you just kind of feel trapped. (P05)

## Discussion

This study sought to understand overweight and obese women’s experiences of PA, diet and QoL during and following pregnancy. Previously, little work has examined overweight and obese women’s experiences, and to our knowledge, we are the first to conduct formative research in women with a BMI > 25 kg∙m^2^ prior to the design and implementation of postpartum lifestyle interventions in the United Kingdom.

Whilst a number of previous investigations have highlighted many PA and nutritional barriers during pregnancy and the postpartum period, (Coll et al., 2017; Doyle et al., 2016; Saligheh et al., 2016; Powell et al., 2007) little work exists in the overweight and obese population. Our findings highlighted a perceived lack of support from medical professionals (e.g., general practitioner and midwife) and discouragement with regards to engaging in PA from friends and family, which agrees with previous research. (Flannery et al., 2018; Harrison et al., 2018; Sui et al., 2013) Sui et al. (2013) conducted semi-structured interviews with 26 overweight pregnant women with the aim of understanding barriers to and enablers of initiating healthy behaviour change during pregnancy. Amongst other barriers, women described prioritising family commitments over their own health, the cost of exercise classes and healthy eating, a lack of support from friends and family, a lack of knowledge of safe exercise during pregnancy and concerns about the safety of the baby as preventing them from engaging in a healthy lifestyle during pregnancy. The results from the current study offer indications that, in the United Kingdom, overweight and obese women experience similar barriers to PA during pregnancy as those residing in Australia. (Sui et al., 2013) Given that overweight and obese women prefer to defer weight management to the postnatal period and view healthy eating as easier to achieve and more important than PA for maternal and infant health, (Goldstein et al., 2021; Weir et al., 2010) future interventions must provide detailed information on the importance of PA and how to exercise safely during pregnancy, and encourage higher levels of support, both from friends and family and the research team. Exercise programmes, specifically, must be affordable and adaptable to fit into women’s time constrained schedules.

As well as describing a range of barriers to PA engagement, women in the current study described numerous issues when attempting to eat healthily during pregnancy. Pregnancy specific barriers such as nausea, in particular morning sickness and being repulsed by certain foods, were identified as preventing healthy eating. Universal barriers included unhealthy cravings, tiredness and a lack or loss of restraint. In high-income settings, little research has focused on understanding specific barriers to healthy eating in pregnant women, especially in those living with overweight and obesity. Blau et al. (2020) conducted a focus group study to understand experiences and understanding of food cravings in pregnant women with mixed BMIs. In concordance with the current work, several participants described that they were using pregnancy as an excuse to eat unhealthily, rationalised by the fact that they were ‘eating for two’. The current work has begun much needed investigations into barriers to healthy eating in overweight and obese pregnant women, however further work in high-income countries is still required. Given the lack of antenatal dietary training provided to medical professionals (Lucas et al., 2014) and a lack of support on how to address pregnancy weight in a non-judgmental manner (Flannery et al., 2019), the delivery of nutritional education programmes is also required with the to aim to support women to improve their dietary behaviours during pregnancy, regardless of their BMI.

The postpartum period is an opportune time to implement long-term healthy lifestyle changes, (Faria-Schützer et al., 2018) due to the fact that women are more aware of their nutrition and bodyweight, (Wilkinson et al., 2015) and are motivated to improve both their own health and that of the baby. (Hanson et al., 2017) Individuals in the present study, however, described a range of perceived barriers to PA engagement and healthy eating, including a lack or loss of routine, time, tiredness and being unable to access appropriate and affordable postpartum exercise classes, which agrees with work by Saligheh et al. (2016). However, Saligheh et al. (2016) do not report the weight status of participants, so we are unable to conclude if these barriers are concurrent across BMI categories or specific to overweight and obese women. In our study, individuals also mentioned prioritising the baby’s health above their own and often regarded classes for the baby as more important and easier to attend as the focus was on the baby, rather than the mother. Women also described a lack of advice from medical professionals and a lack of support from friends and family as preventing postpartum PA engagement. These findings agree with previous work whereby new mothers consider parenting as the most important responsibility following childbirth, (Paskiewicz, 2001) and a lack of advice from professionals regarding appropriate exercise programs has been identified as a barrier to postpartum participation in a cohort of women where 52.7% of them were overweight or obese. (Evenson et al., 2009).

There exists a lack of work exploring specific barriers to postpartum healthy eating practices, although our findings still offer substantial agreements with reasons for women declining the invitation to participate in a postpartum lifestyle intervention (exercise and diet), which included time pressures, lack of support from family and friends and prioritising other life commitments over their own health (Carter-Edwards et al., 2009). Women in the current study also described using breastfeeding as an excuse to eat more, which agrees with the results by Lyons et al. (2019) who described that obese women perceive the need to consume more calories in order to maintain milk supply than non-obese women. (Lyons et al., 2019) Given that unfavourable maternal and child clinical outcomes relate linearly to BMI, (Stubert et al., 2018) it is perhaps not surprising that overweight and obese women in the current study identity medical complications as the most common perceived barrier to postpartum exercise. Previously, medical limitations and recovery from caesarean section were identified as a main barrier to exercise engagement in only 4.7% of a mixed BMI postpartum population. (Evenson et al., 2009) Therefore, healthcare professionals must work more closely with overweight and obese women to support and encourage a safe and timely return to, or initiation of, PA and exercise following the recovery from childbirth.

In the current study, participants described that exercise and healthy eating had a positive influence on QoL. A lack of sleep, loneliness and a loss of freedom since the baby had arrived were identified as factors that negatively influenced QoL. Jeong et al. (2021) conducted a questionnaire study exploring factors that affect QoL in the six weeks following childbirth in South-Korean women, and also found that tiredness and mental health influenced postpartum QoL. Further, postpartum women describe that marital intimacy and high relationship satisfaction, breastfeeding adaptation, sleeping patterns, and occupation impact on their QoL (Valla et al., 2022; Jeong et al., 2021). The results from the current interview study and recent previous questionnaire studies (Valla et al., 2022; Jeong et al., 2021) contribute to a better understanding of the factors influencing postpartum QoL and the need to identify sub-groups at risk of low postpartum QoL that may require further support, however until now, no work has focused on understanding the specific experiences of women living with overweight and obesity.

### Practical Applications

Herein, we have provided a comprehensive understanding of perceived universal and pregnancy and postpartum barriers to a healthy lifestyle in women living with overweight and obesity in the United Kingdom. This information is imperative prior to designing and delivering lifestyle interventions, as such we would encourage others to apply the findings to the design of postpartum weight management programmes for women living in similar settings and with comparable background characteristics.

Only a small number of previous studies have completed formative work prior to implementing lifestyle interventions in postpartum women and have provided mixed results. For example, following a needs assessment of the barriers to weight-related health behaviours, Graham et al. (2016) demonstrated no significant difference in the proportion of women who experienced excessive GWG when comparing intervention (48.1%) and control (46.2%; p = 0.12) groups. The authors concluded that the low usage of the behaviour change tools (46.1%) in the intervention group and the similarity between the control and intervention treatments may explain the absence of differences between groups. In other areas, formative work carried out prior to the implementation of interventions has proven more effective. Danaher et al. (2012) included formative work with focus group participants and usability testers that contributed towards the design of a web-based intervention aimed at ameliorating the symptoms of postpartum depression. (Danaher et al., 2012) Results from the intervention revealed that 55% of participants met the criteria for minor or major depression prior to the program and at the post-test 90% no longer met the criteria. (Danaher et al., 2013)

In the overweight and obese postpartum population, it is evident that formative work is still required to identify the necessary tools to promote significant weight loss and improve both maternal and infant outcomes. Based off the results of the current study, we have created a list of suggested practical applications (see Tables 8 and 9) to assist researchers when designing and delivering future postpartum lifestyle interventions in overweight and obese women. We recommend that, to effectively intervene and influence the cycle of obesity in the postpartum and inter-pregnancy period, a holistic understanding of pregnancy and postpartum weight trajectories and associated characteristics is crucial to support and promote healthy behaviours during this time. (Muñoz-Manrique et al., 2022) Furthermore, medical professionals should utilise this information in primary healthcare settings when encouraging women to engage in healthy lifestyle behaviours before, during and following pregnancy.

**Table 8:** Postpartum exercise barriers and suggested practical applications for future interventions

Exercise Barriers (Postpartum)	
Theme	Suggested Practical Application
Medical Complications	Individualised, incremental increases in exercise levels/intensity throughout an intervention. Recruitment following 6-8-week postpartum health check/received approval from general practitioner to resume physical activity following the birth.
Convenience	At home exercise programmes.
Routine	Emphasise the importance of exercise for maternal health and support mothers in incorporating exercise into daily routines. Design exercise programmes whereby sessions can be completed in short time periods and incorporated into busy routines.
Support	Encourage support at home from family and friends. Include other forms of support (e.g. technology) through Facebook/WhatsApp groups whereby mothers can support each other.
Time	Design exercise programmes whereby sessions can be completed in short time periods and at different times of the day.
Childcare	At home exercise programmes where the baby can be incorporated into exercise sessions/sessions can be completed during, for example, nap time or when the partner is home/available for childcare.
Tiredness	Encourage women to complete sessions/walks when they feel less tired/able. Emphasise the importance of walking and exercise for maternal health and provide consistent support to encourage an active lifestyle.
Motivation and enjoyment	Include a variety of exercises, and types of exercises (endurance and strength) to reduce boredom and increase enjoyment.
Cost	Free sessions.
Breastfeeding	Encourage women to develop a plan to exercise around the breastfeeding routine. Exercising at home also allows the mother to attend to breastfeeding needs.
Confidence	At home exercise sessions, without the judgement or suspected judgement of other women in group exercise classes. Group support (through technological means) to encourage increases in self-confidence.

**Table 9:** Postpartum nutrition barriers and suggested practical applications for future interventions

Nutrition Barriers (Postpartum)	
Theme	Practical Application
Time	Include quick recipe suggestions as part of the nutrition intervention and, where possible, encourage childcare support from family members/friends to allow time for food preparation.
Tiredness	Encourage women to employ a range of behavioural techniques (e.g. batch cooking when not tired) so as to stay on track when feeling tired.
Routine	Support women to develop a daily/weekly routine whereby time is allocated to, for example, planning the weekly food shop and batch cooking in advance.
Support	Encourage support at home from family and friends. Include other forms of support (e.g. technology) through Facebook/WhatsApp groups whereby mothers can support each other on the programme.
Motivation and enjoyment	Utilise technological support to increase motivation and encourage other women to provide recipe suggestions/healthy eating tips on social media groups (e.g. Facebook/WhatsApp).
Breastfeeding	Provide education on the caloric requirements of breastfeeding as part of the intervention.
Restraint	Emphasise the importance of a healthy diet and motivate women to develop restrained eating behaviours to encourage healthy maternal and offspring outcomes.

## Conclusion, Limitations and Future Directions

Our study included women who were 12–52 weeks postpartum, predominantly of white ethnicity and mostly educated to at least degree level. Future research should look to engage with women from ethnic minority groups who are less educated and at set postpartum time points to understand any similarities and differences in women’s experiences through the postpartum journey. Interviews were also only conducted at one time point. In future, an understanding of women’s perceived barriers at different time points will enable an understanding of the prominence of certain barriers throughout the postpartum period. The range in interview lengths may also indicate that some participants were not fully engaged or had few, or no, perceived barriers to a healthy lifestyle during and following pregnancy. In some instances, a follow-up interview would have been useful. Finally, examining the relationship between participants’ barriers to a healthy lifestyle and engagement with local lifestyle support services would be useful to identify any areas within such services that could be improved upon to encourage healthy long-term outcomes.

The current study was novel in design whereby we have gained valuable insights into overweight and obese postpartum women’s experiences of PA, diet and QoL during and following pregnancy, and provided suggestions for the practical implementation of future lifestyle interventions. Overweight and obese women appear to encounter several universal barriers experienced by the general population and describe similar challenges to normal weight pregnant and postpartum women when attempting to engage in a healthy lifestyle. Based on our findings, overweight and obese postpartum mothers do not describe any unique barriers to a healthy lifestyle, other than medical complications preventing exercise engagement. Both the universal and postpartum specific barriers should be considered in future intervention work. We intend to use the results from the current study along with previous work to inform the design of our future lifestyle intervention studies delivered in women with overweight and obesity following childbirth.

### Electronic supplementary material

Below is the link to the electronic supplementary material.

Appendix 1: Interview guide

## Data Availability

All data generated or analysed during this study are included in this published article (and its supplementary information files).
