# Supplementary material for: Experiences of Physical Activity, Healthy Eating and Quality of Life During and Following Pregnancy in Overweight and Obese Postpartum Women
Source: Matern Child Health J. 2023 Jun 14;27(11):1968–80. doi: 10.1007/s10995-023-03684-7 (PMC10564817; doi:10.1007/s10995-023-03684-7)
Supplement: Supplementary file 1 — Appendix 1: Interview guide [file 10995_2023_3684_MOESM1_ESM.docx]

**Interview Guide**

Thank you for agreeing to take part in the interview today. I want to remind you that you have the right to withdraw from the study at any time and if there are any questions that you don’t want to answer, you don’t have to. I’ll be asking you about your experiences with physical activity [exercise] and food in general and before, during and after pregnancy and I shall be recording the whole interview. The interview should last about an hour. Are you happy for the interview to be recorded and have you got any questions before we get going?

*The overall purpose of this interview, in line with narrative research, is to seek stories from the participants. In response to these stories some questions might be adapted and further probes used.

Introductory Questions

- Did the birth go to plan? / Was it what you expected?
- How are you feeling since the birth?

Main Questions (Physical Activity)

- When I say the words “physical activity” what comes to mind?
- What is your overall experience with physical activity? Do you have good or bad memories?
- Probe: positive, negative, growing up, recently
- Can you tell me any stories related to physical activity? Recently, before or during your pregnancy?
- Can you tell me about your physical activity experiences before pregnancy?
- What was your physical activity like in each of your trimesters? Were there any changes as you progressed through your pregnancy?
- What is your physical activity like now?

Main Questions (Nutrition/Food)

- When you think about “nutrition/food” what comes to mind?
- Can you tell me any stories that stand out related to your diet/what you eat?
- Probe: recently, younger, during pregnancy
- What are your overall experiences with food?
- Probe: positive, negative
- Are you able to tell me about your dietary experiences at different stages during your pregnancy?
- Probe: trimester changes, cravings, amounts
- What are your overall thoughts about what you eat?
- What changes in your diet have you noticed since the birth?

Summary Questions

- How does food and exercise affect you in general?
- Probe: emotions, thoughts, feelings, quality of life
- How does physical activity and food relate to your quality of life?
- How has your quality of life been [changed] since the birth?
- Is there anything else you want to say or tell me about?

Thank you so much for participating in the interview.
